# Simulation as a Learning Tool in the Oncology Setting

**Published:** 2014-05-01

**Authors:** Patricia C. Simmers

**Affiliations:** The Ohio State University Wexner Medical Center, Columbus, Ohio

Simulation-based education in medicine represents a teaching paradigm shift. David Gaba, associate dean for Immersive and Simulation-Based Learning at Stanford University School of Medicine, describes simulation as a technique, not a technology, that recreates real patient experiences and gives participants the opportunity to learn concepts, develop skills, and practice without causing harm to the patient (Gaba, 2012). Research has shown simulation improves learning for both medical and nursing students in many different clinical domains (Gaba, 2012). Simulation can involve use of high-fidelity mannequins, role-playing, storytelling, or standard patient actors. Currently, there are no templates on how to do simulation training, but varied methods are used in medical schools, schools of nursing, and health-care organizations (Patow, 2005).

Okuda et al. (2009) indicate a current trend of patient concern regarding being "practiced on" by medical or nursing students in the health-care setting. Simulation offers a vehicle for clinical outcome improvement along with a reliable learning outcome measurement. Participants in the simulation experience learn principles of teamwork and communication. Simulation can effectively assess knowledge gaps and provide a safe and supportive learning environment.

## Background

In nursing education, there is rarely a substantial focus placed on teaching oncology content. Historically, in nursing programs, content is presented on fundamental nursing practice, medical surgical nursing, maternal child, and mental health nursing. Various frameworks being used in schools of nursing do not focus in any length on teaching oncology concepts or topics. This article describes a nurse faculty program to train newly graduated, novice RNs for the role of an oncology nurse using simulation as a learning tool. The author of this article participated in the design and implementation of the program.

The impetus for this project involved a partnership between the College of Southern Nevada (CSN) School of Nursing, the Nevada Cancer Institute, a Department of Labor Grant, and the Nevada Workforce Development to train graduates from the CSN associate degree nursing program into the field of oncology nursing. Nurses attended monthly simulation sessions for a total of 18 months and participated in high-fidelity oncology emergency simulations with a two-person team consisting of an oncology-experienced nursing faculty member and a school lab/simulation technician. Ten oncologic emergencies were written, designed, and implemented. Nurse participation involved mostly novice level nurses with little to no oncology experience.

## Project Goals and Methods

The goals of the project in relationship to the graduate nurses participating were to teach content regarding 10 oncology scenarios commonly seen in the oncology population. This teaching included experiential learning through high-fidelity simulation, debriefing, evaluation, posttest questions, PowerPoint presentations, and videos. At the conclusion of 1 year, graduate nurses would have the opportunity to sit for the OCN exam, the fee for which would be covered by their employer. Graduate nurses participating in the project also gained valuable experience working with oncology patients and then could have the opportunity to seek an RN position in oncology once their internship was completed.

## Simulation Design

Scenarios developed and evolved using the real-life experiences of the oncology nurse faculty member or were gleaned from published oncology case studies. Oncologic emergencies to be studied included anaphylaxis, tumor lysis syndrome, bowel obstruction, hypercalcemia, neutropenic sepsis, disseminated intravascular coagulation (DIC), cardiac tamponade, increased intracranial pressure, spinal cord compression, and syndrome of inappropriate antidiuretic hormone (SIADH). Novice nurses were not necessarily informed about which oncology emergency was occurring prior to simulation. They were tasked to use their knowledge and assessment skills to determine the problem and to implement nursing interventions to ensure the best patient outcomes.

Groups of eight students participated in any given session. Four students participated in the simulation, while the other four were observers, thus having the opportunity to assess their peers’ competency in performing the simulation activity. The mannequin was programmed to have vital signs, voice, or sound, and nurses played the role of physician, charge nurse, staff nurse, and/or family member. Each participant had a dialog sheet that they followed to act in the scenario. Most scenarios included lab results or test reports that needed to be evaluated.

The nursing faculty took anecdotal notes after each simulation activity and shared the analysis with the appropriate stakeholders. When each scenario was developed, journal articles relevant to the topic were used to ensure that evidence-based practice was current. The articles were also provided to each graduate nurse for discussion after debriefing. This practice enhanced learning and understanding of any new concepts acquired during the simulation.

## Simulation Technology

Minimal research has been done on the effectiveness of using simulation in nursing education. Today’s health-care system emphasizes the role of providing "accurate and safe care to patients" (Sanford, 2010). Simulation is emerging as an alternative teaching strategy, as it encompasses the use of theory, assessment, technology, pharmacology, and clinical skills (Rauen, 2004). Through experiential methods, oncology nurses learn the appropriate and evidence-based way to treat patients when an oncologic emergency occurs.

Teaching with technology is an alternative method to face-to-face education. It is not necessarily better or worse; it is just different (Bates & Poole, 2003). Many of the participating nurses felt pushed out of their "comfort zone" as they were propelled into unknown situations where they had to determine the best route to take. Through the use of high-fidelity simulation, novice nurses had the opportunity to be exposed to varied oncologic emergencies and were evaluated and debriefed on their use of nursing knowledge and critical thinking pathways while participating in the simulation. Quality and Safety Education for Nurses (QSEN) competencies provided the basis for assessment and evaluation of whether participants demonstrated knowledge and competency in treating the oncology emergency.

Current trends in nursing education and health care place a high importance on achievement and flawlessness in performing any nursing skill or intervention. Simulation allows participants to challenge themselves and make errors in a safe and supportive environment with no harm being done to a live patient (Arafeh, Snyder Hansen, & Nichols, 2010). The oncology simulations done with novice oncology nurses were performed in a way that did not place significance on achievement, but focused instead on learning and pushing each participant toward a higher level of critical thought and competence.

## Instructor Methods/Debriefing

Debriefing is a critical component in the simulation experience (Arafeh, Snyder Hansen, & Nichols, 2010). The debriefing session following each oncology simulation was implemented as a teachable moment. One goal was to encourage participants to use critical thinking and limited nursing experience—along with recent fundamental nursing knowledge acquired in nursing school—to assess present knowledge and nursing competency.

The debriefing period is invaluable, as it is the portion of simulation during which analysis and perspective emerge as one. Participants are given the opportunity to reflect and discuss their clinical performance in meeting scenario objectives that are aligned with current oncology nursing practice and evidence-based guidelines. Using a real-life basis for the scenarios proved to be most beneficial to the novice nurses.

Upon completion of the simulation scenario, participants were evaluated by their instructor. Background information was provided on the specific oncologic emergency in a teachable, nonthreatening way through handouts, group question-and-answer sessions, videos, and student participation. As mentioned previously, recent journal articles were provided to the novice nurses as "take-home" items that highlighted current evidence-based practice related to the oncologic emergency being studied that day. Participants were able to discuss what had been done "right" and what could have been done better. Arafeh and colleagues (2010) concur on the importance of ensuring constructive feedback during these sessions using open group discussion in an environment supporting open communication and group learning methods.

## Measurement Outcomes

Project outcomes were measured through graduate nurse self-evaluation and instructor evaluation of performance using QSEN guidelines as a framework. Domains included patient-centered care, teamwork and collaboration, evidence-based practice, quality improvement, safety, and informatics. Each domain included knowledge, skills, and attitudes relevant to the particular scenario or oncologic emergency. Peers and faculty members assessed participants with respect to whether the necessary performance requirements were appropriately achieved. See the Appendix at the end of this article for more details on a sample scenario and the associated objectives and assessment rubrics.

Graduate nurses’ views of simulation were not consistent at any given time. Some experienced much angst and did not want to be "on stage." Others stated they learned best from the debriefing moments that occurred after the simulation completed. Teamwork was often viewed when a strong nurse was placed with a weaker one who needed support in making the appropriate decision in patient care. Faculty members noticed that after four simulation experiences, most nurses experienced less angst and enjoyed the learning experience much more.

## Drawbacks and Merits of Simulation

There are many advantages and disadvantages to teaching with simulation. One disadvantage of simulation is that it requires significant preparation, support, and buy-in from those involved in the activity (Okuda et al., 2009). Nursing faculty members never exposed to the concept of high-fidelity simulation are often hesitant to facilitate activities due to lack of past exposure and minimal training. In addition, resources in nursing schools are not always optimal. In the experience described in this article, a two-person team had to coordinate the whole process, which proved to be quite challenging. Neither member had had formal training in simulation prior to participating in this project. Having QSEN competencies and simulation examples available prior to the start of the project provided a framework to work from. Graduate nurses participating in the oncology simulations often felt that they were on stage or felt uncomfortable not knowing what was expected of them. They believed that it would be somehow held against them if they could not attain what was expected of them by faculty. The nurses lacked clinical experience in oncology and had no oncology hospital experience, which placed them at a disadvantage.

Despite these disadvantages, the advantages to simulation are numerous. Being in a safe environment where making mistakes is a part of learning decreases the risk of causing harm to a live patient. Research indicates that simulation enhances learning by improving a nurse’s skills and providing educators with a method to assess a student’s understanding of the concepts being taught (Patow, 2005).

## CONCLUSIONS

High-fidelity simulation is evolving and emerging as an alternative or adjunct method to clinical experience in many medical settings, including oncology. Methods may vary among individuals, but the essential first steps in developing a simulation training program include coming up with a case study that revolves around a medical concept, determining course outcomes and course expectations for the learner, and structuring evaluation tools that assess whether outcomes have been achieved.

Although the program described in this article focused on nurses, the benefits of simulation training can also be applied to advanced practitioners in oncology: nurse practitioners, clinical nurse specialists, and physician assistants. These clinicians could benefit from an adjunct learning strategy such as simulation because practice and repetition usage in simulation aid in acquiring medical expertise and comprehension.

The shortage of medical professionals will continue, and the lack of clinical experiences for novice oncology nurses will endure. The aging of our nursing workforce impels educators to come up with new methods to train our future oncology nurses in a safe, effective environment. Simulation provides a safe method to teach, engage, and build confidence in novice nurses as they transition to a higher level of understanding of how to respond to oncologic emergencies.

**Appendix:**
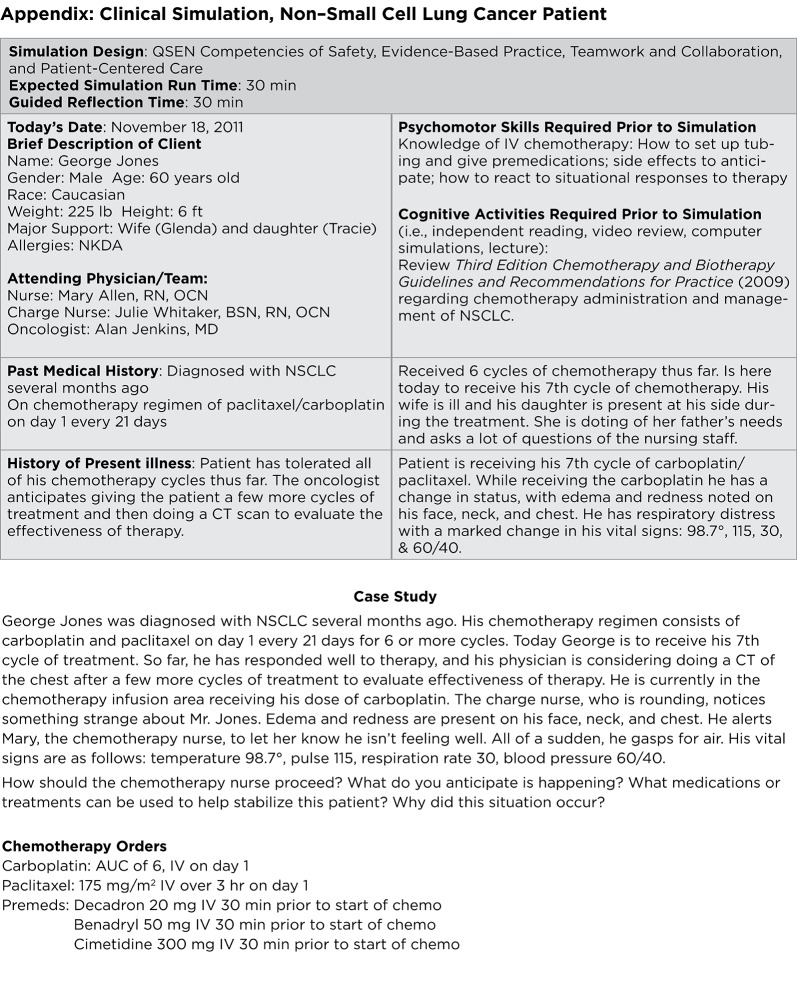
Clinical Simulation, Non–Small Cell Lung Cancer Patient

**Figure:**
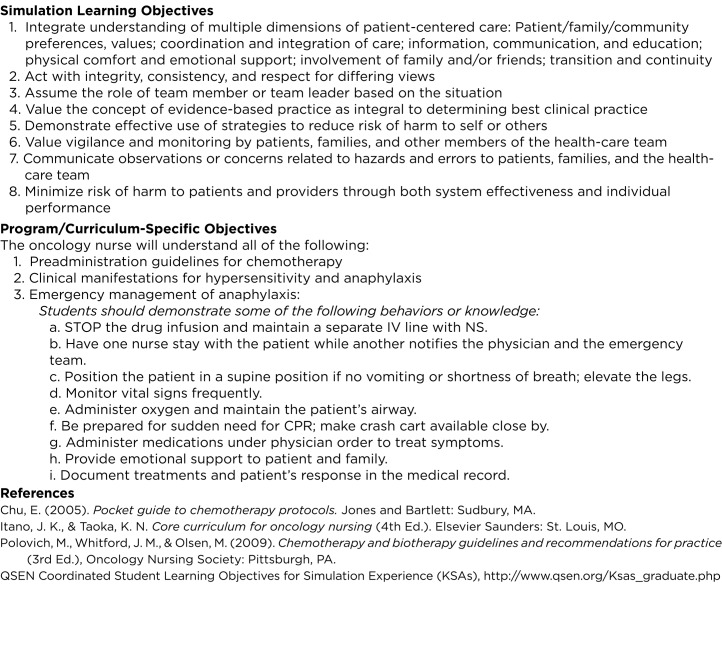
Case Study (continued)

**Figure:**
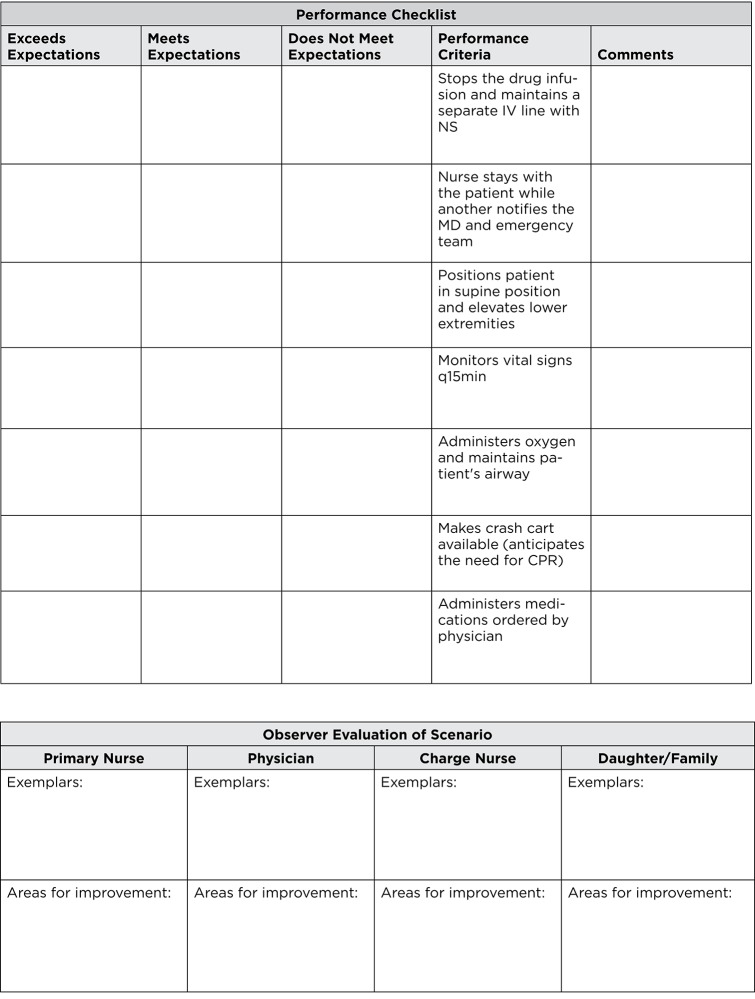
Performance Checklist
